# Haemobilia Secondary to Spontaneous Cystic Artery-Gallbladder Fistula: A Unique Gastrointestinal Anomaly

**DOI:** 10.7759/cureus.9457

**Published:** 2020-07-29

**Authors:** Sudeep Acharya, Indraneil Mukherjee, Shamsuddin Anwar, Gloria Lan, Abhishek Polavarapu

**Affiliations:** 1 Internal Medicine, Northwell Health-Staten Island University Hospital, Staten Island, USA; 2 Surgery, Northwell Health-Staten Island University Hospital, Staten Island, USA; 3 Gastroenterology, Northwell Health-Staten Island University Hospital, Staten Island, USA

**Keywords:** haemobilia, clinica gastroenterology, emergent general surgery

## Abstract

Spontaneous cystic artery-gallbladder fistula is an extremely rare condition described in the medical literature. We have found two articles in the literature describing fistula formation between the cystic artery and gall bladder. In this report, we present a unique case of a cystic artery-gall bladder fistula that resulted in massive gastrointestinal bleeding through cystic artery erosion and was adequately managed with coil embolization.

## Introduction

Haemobilia is a rare cause of upper gastrointestinal bleeding usually secondary to an iatrogenic or trauma-induced abnormal cystic artery and biliary tract connection. Spontaneous cystic artery-gallbladder fistula has rarely been discussed in the medical literature [1]. In this case report, we describe a case of spontaneous cystic artery-gall bladder fistula resulting in massive gastrointestinal bleeding. The patient was successfully managed with cystic artery coil embolization.

## Case presentation

A 62-year-old female with a pertinent past medical history of HIV on antiretroviral therapy, dyslipidemia and glaucoma initially presented to the emergency department for evaluation of abdominal pain and bright red blood per rectum. The abdominal pain had started two days before the presentation; it was described to be generalized and associated with bloating. She also endorsed having three bowel movements with bright red blood per rectum at home. She had undergone outpatient colonoscopy screening the previous year, which had been reported to be normal. She denied any history of adenoma, polyps, and personal or family history of gastrointestinal-related cancers. She only had a pertinent surgical history of appendectomy. Her drug history was significant for the daily consumption of non-steroidal anti-inflammatory drugs once in the past two years for chronic right hip pain.

**Figure 1:**
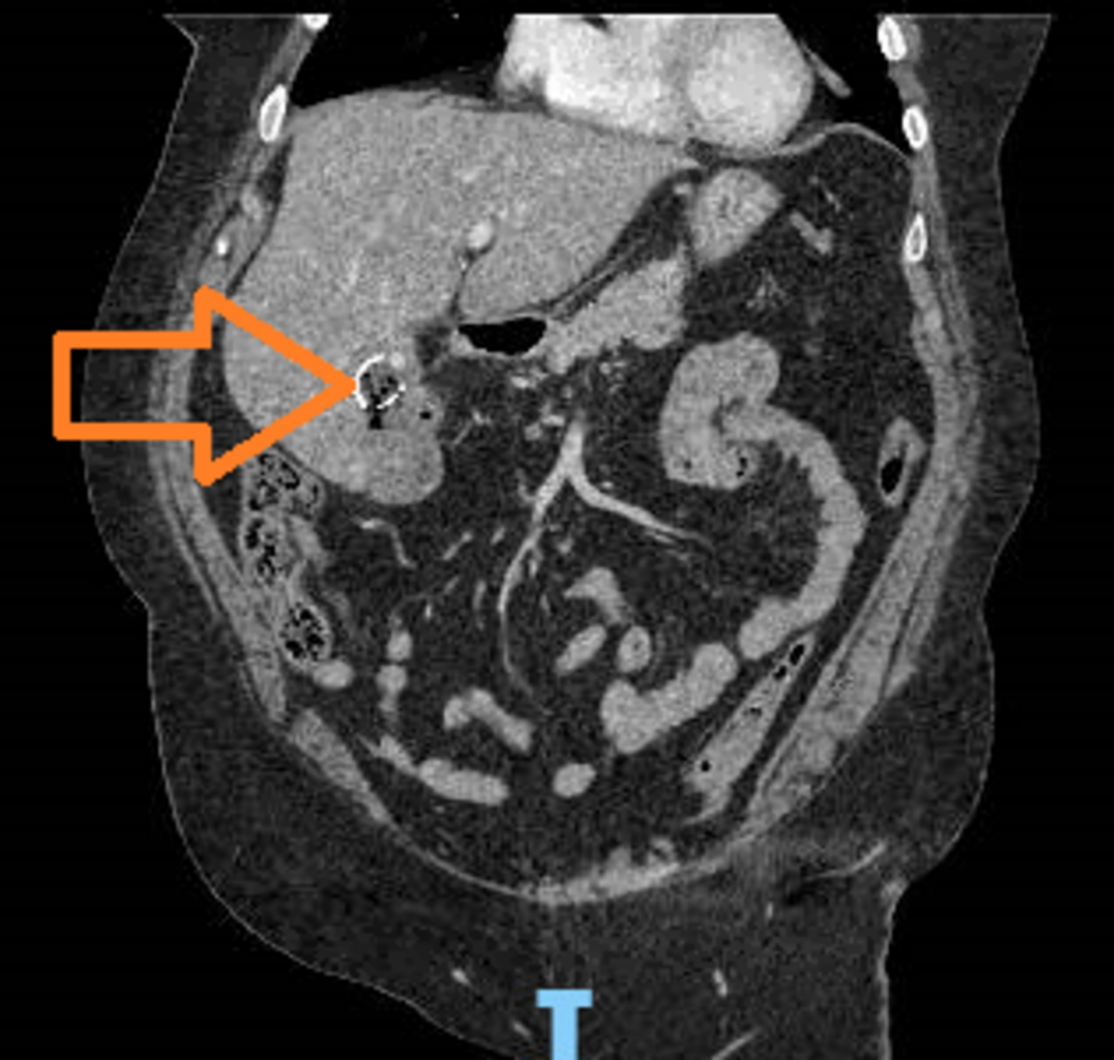
CT of the abdomen Arrowhead shows the porcelain gallbladder CT: computed tomography

Initially, in the emergency department, the patient remained hemodynamically stable. The liver function tests showed mildly elevated mixed pattern with alkaline phosphatase of 313 IU/L (normal range: 44-147 IU/L), aspartate aminotransferase of 92 Units/L (normal range: 17-59 Units/L), and alanine aminotransferase of 65 Units/L (normal range: 0-35 Units/L). Given her history of abdominal pain, a CT scan was obtained, which demonstrated porcelain gall bladder with suspicion of a fistulous tract between her gallbladder, colon, and duodenum (Figure 1).

**Figure 2:**
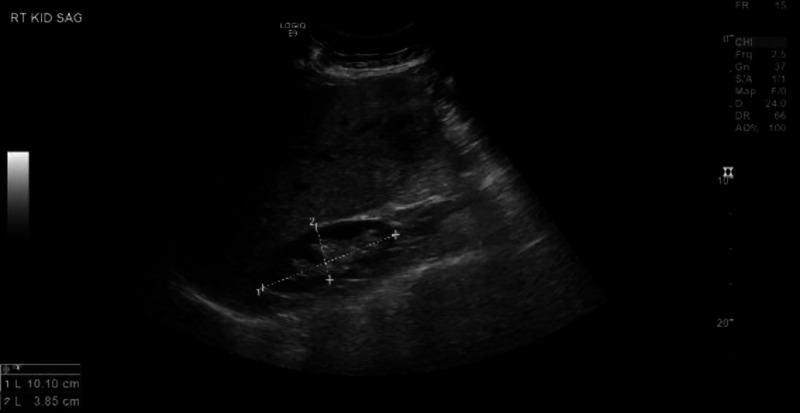
Ultrasound of gall bladder The dotted lines demonstrate the dimensions of stone inside the gall bladder

Feeding was held, and she was kept on intravenous fluids, prophylactic antibiotics (ciprofloxacin and metronidazole), and proton pump inhibitors. She was evaluated by general surgery and gastroenterology and was planned for esophagogastroduodenoscopy (EGD) and colonoscopy the next day. Ultrasound of the abdomen was additionally obtained to visualize the hepatic structures, which showed mild hepatomegaly with suspected stable 2.5-cm gall stone and negative Murphy’s sign, ruling out cholecystitis. The common bile duct was noted to be normal with 4 millimeters in size (Figure 2).

On the second day of hospitalization, she developed an episode of massive bleeding per rectum after which her hemoglobin dropped from a baseline of 9.5 to 6.9. She was emergently resuscitated with intravenous fluids and four units of packed red blood cells and was taken for emergent EGD. There was no evidence of a cholecystoduodenal fistula on EGD. It showed multiple non-bleeding clean based ulcers in the antrum, body, and fundus of the stomach of variable sizes (ranging from 8 millimeters to 2 centimeters). In the second portion of the duodenum, a bright red blood clot was noted, which was not dislodged upon washing. As bleeding was noted to be dripping from the ampulla, there was high suspicion for haemobilia likely due to hepatic artery fistula into the gall bladder. Intervention radiology was immediately consulted and, upon reviewing the CT scan again, there was high suspicion for cystic artery pseudoaneurysm. However, when the patient emergently underwent a hepatic angiogram, cystic artery fistula with the gallbladder was confirmed. Subsequently, cystic artery coil embolization was performed to control the bleeding vessel. The patient was transferred back to the intensive care unit for further monitoring.

**Figure 3:**
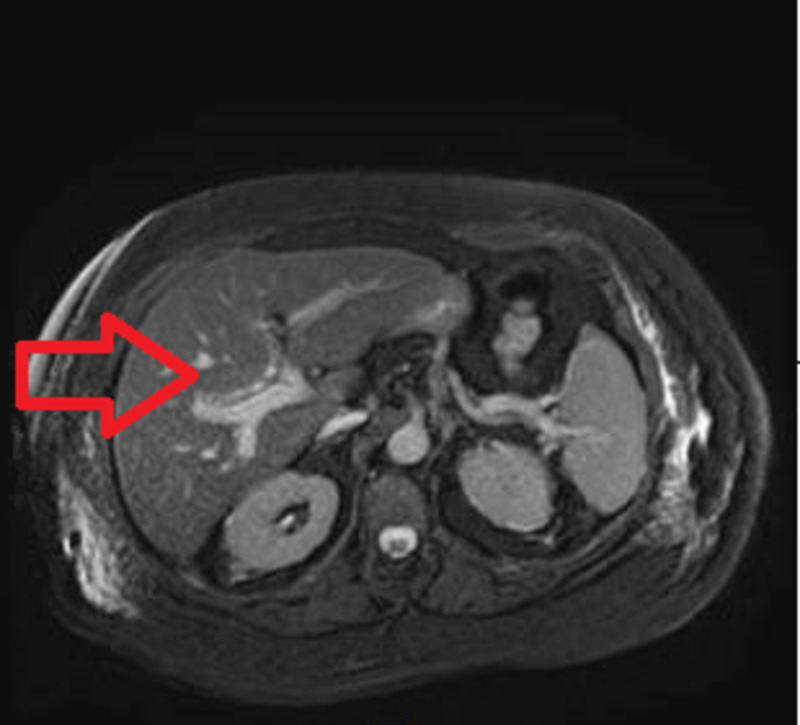
MRCP of the abdomen The arrowhead demonstrates the biliary tract with no fistulous formation to the duodenum MRCP: magnetic resonance cholangiopancreatography

After reviewing the imaging studies and extensive discussion with surgical, radiology, and gastrointestinal teams, it was concluded that the diagnosis of the gallbladder-duodenal fistula was misleading due to the thickened gallbladder wall. In order to further improvise on the anatomical structural findings, MRI of the abdomen with and without contrast was obtained, confirming no evidence of any fistulous tract between gall bladder and duodenum (Figure 3). Gall bladder wall calcification that was noted may have represented gall bladder stone versus calcification of the gall bladder wall. No gallbladder mass was seen, and her symptoms completely resolved after the radiologic intervention. She was able to tolerate a regular diet with no gastrointestinal symptoms. Her bowel movements were regular with no further evidence of bleeding. Her hemoglobin remained stable, and she did not require any more transfusion and was subsequently discharged home.

Currently, she is recovering at home and will continue to follow up with us for possible elective cholecystectomy for finding the porcelain gall bladder, which may represent the underlying malignancy.

## Discussion

Spontaneous fistula formation between the cystic artery and gall bladder has rarely been reported in the medical literature. As seen in our patient, it may result in a rare cause of haemobilia [1-3]. Haemobilia comprises approximately 6% of all the causes of acute gastrointestinal bleeding [4]. The most common etiology of haemobilia is iatrogenic (surgical intervention), inflammatory process (such as cholecystitis), or traumatic, which results in an abnormal connection between the cystic artery and biliary tract [5-8].

Identifying vascular-biliary fistula can pose a diagnostic challenge. In our clinical case, the initial CT scan of the abdomen falsely suggested possible fistula formation between gall bladder, colon, and the duodenum. However, on EGD, active bleeding from the ampulla of Vater was clearly noted in the duodenum. No abnormal tracts suggestive of biliary-enteric fistula were visualized during the endoscopic procedure. A prompt angiogram was performed by the intervention radiology, which effectively identified the bleeding vessel to be a cystic artery and the patient underwent coil embolization. Although aneurysm and pseudoaneurysm of the cystic artery are considered important differentials leading to such vascular-biliary fistula, no such abnormality was noted in the angiogram. Therefore, the cystic artery was coiled, which successfully controlled the bleeding [9,10].

The exact mechanism of the spontaneous cystic artery and biliary tract fistula is not properly defined. However, it can be suggested that the inflammatory process following the stone formation in the biliary tract and mechanical pressure of the stone may have resulted in adhesion formation with the anatomical structures in proximity, promoting fistula formation [11].

As the suspicion for cystic artery erosion resulting in active bleeding in the gastrointestinal tract through the cystic artery-gallbladder fistula was considered very high, cystic artery coil embolization was performed. The bleeding was successfully stopped, resulting in the stabilization of the patient. The MRI studies performed later confirmed the suspicion for cystic artery-gall bladder fistula by ruling out any suspicious abnormality in the biliary tract. As limited information is available in medical literature for the treatment of spontaneous cystic artery-gallbladder fistula, we believe our case with coil embolization will provide a unique example in the management of such a clinical entity [12]. Given the porcelain texture of the gall bladder with cholelithiasis evident in the imaging studies of our patient, she is currently being considered for an elective cholecystectomy after she recovers from this event.

## Conclusions

A spontaneous cystic artery-gall bladder fistula is a unique clinical condition and hence its diagnosis is challenging. It may cause massive gastrointestinal bleeding as observed in our clinical scenario. It should be considered as a differential for haemobilia. From our clinical scenario, it can be suggested that minimally invasive treatment with coil embolization can constitute the best strategy for management, thereby securing the hemodynamic stability of the patient. It may also be used as a bridge to surgical resection of the gall bladder if deemed necessary. Further review of medical literature is warranted to understand the abnormal fistulous formation between vasculature and gall bladder.
